# Gastric bypass surgery weight loss-independently induces gut Il-22 release in association with improved glycemic control in obese Zucker fatty rats

**DOI:** 10.1016/j.metop.2022.100212

**Published:** 2022-10-02

**Authors:** Florian Seyfried, Rebecca Springer, Annett Hoffmann, Maximilian Gruber, Christoph Otto, Nicolas Schlegel, Mohammed K. Hankir

**Affiliations:** Department of General, Visceral, Vascular and Pediatric Surgery, University Hospital Wuerzburg, Wuerzburg, 97080, Germany

## Abstract

**Background:**

Roux-en-Y gastric bypass surgery (RYGB) improves glycemic control in individuals with severe obesity beyond the effects of weight loss alone. To identify potential underlying mechanisms, we asked how equivalent weight loss from RYGB and from chronic caloric restriction impact gut release of the metabolically beneficial cytokine interleukin-22 (Il-22).

**Methods:**

Obese male Zucker fatty rats were randomized into sham-operated (Sham), RYGB, and sham-operated, body weight-matched to RYGB (BWM) groups. Food intake and body weight were measured regularly for 4 weeks. An oral glucose tolerance test (OGTT) was performed on postoperative day 27. Portal vein plasma, systemic plasma, and whole-wall samples from throughout the gut were collected on postoperative day 28. Gut *Il-*22 mRNA expression was determined by real-time quantitative PCR. Plasma Il-22 levels were determined by enzyme-linked immunosorbant assay (ELISA).

**Results:**

RYGB and BWM rats had lower food intake and body weight as well as superior blood glucose clearing capability compared with Sham rats. RYGB rats also had superior blood glucose clearing capability compared with BWM rats despite having similar body weights and higher food intake. *Il-*22 mRNA expression was approximately 100-fold higher specifically in the upper jejunum in RYGB rats compared with Sham rats. Il-22 protein was only detectable in portal vein (34.1 ± 9.4 pg/mL) and systemic (46.9 ± 10.5 pg/mL) plasma in RYGB rats. Area under the curve of blood glucose during the OGTT, but not food intake or body weight, negatively correlated with portal vein and systemic plasma Il-22 levels in RYGB rats.

**Conclusions:**

These results suggest that induction of gut Il-22 release might partly account for the weight loss-independent improvements in glycemic control after RYGB, and further support the use of this cytokine for the treatment of metabolic disease.

## 1 Introduction

The global incidence of obesity, defined as having a body mass index (BMI) of > 30–35 kg/m^2^, has almost tripled since 1975, with recent estimates that 650 million of the world's population is obese [1]. In the United States, it is predicted that 50% of adults will be obese by 2030, while 25% will be severely obese, defined as having a BMI > 35 kg/m^2^ [2]. Along with high BMI, the global incidence of obesity-associated comorbidities such as type 2 diabetes continues to rise too, with 480 million adults diagnosed with the condition in 2021 contributing to over 6 million deaths worldwide [3]. As well as negatively impacting health and mortality, obesity and type 2 diabetes place a major socioeconomic burden on governments. In the United States, it is predicted that by 2030 obesity will cost $70 billion in healthcare annually [4], and that type 2 diabetes will have incurred a total healthcare cost of more than $1 trillion [3].

Despite significant progress in the development of improved body weight- and blood glucose-lowering drugs such as the stable glucagon-like peptide 1 (GLP-1) analogue semaglutide, bariatric surgeries remain the gold-standard treatment option for severe obesity [5]. Roux-en-Y gastric bypass (RYGB) is one of the most frequently performed and effective bariatric surgeries, and sustainably causes 30–40% weight loss in individuals with severe obesity in association with marked improvements in glycemic control [6]. Interestingly, some RYGB patients discontinue their diabetes treatments before any changes in body weight, which has led to the hypothesis that surgery-specific mechanisms contribute to this effect [7]. Studies using rodent models of RYGB have further substantiated the idea that RYGB improves glycemic control independently of weight loss [[8], [9], [10]]. Potential mediators that enhance pancreatic beta cell function and/or insulin sensitivity after RYGB include gut hormones (like GLP-1) and bile acids, circulating levels of which are markedly increased by the procedure [11]. However, studies using knockout mice suggest that additional factors contribute to the beneficial metabolic effects of RYGB [12,13]. Better characterization of these factors may assist in the development of improved non-invasive alternatives to bariatric surgery.

Interleukin-22 (Il-22) is a 180 amino acid cytokine belonging to the Il-10 cytokine family that was first discovered in mouse BW5147 lymphoma cells treated with Il-9 [14]. Subsequent studies revealed that Il-22 is released from various gut innate lymphoid cells and CD4^+^ T helper cells during infection to promote host defense by binding to the 1l-10R2 beta chain-Il-22R1 alpha chain receptor complex expressed in gut epithelial cells [15]. In 2014, Wang et al. provided evidence for a novel role of Il-22 in promoting metabolic health [16]. Il-22R1-deficient mice developed obesity on a high-fat diet as well as glucose intolerance and insulin resistance [16]. Conversely, chronic exogenous recombinant Il-22 treatment caused weight loss and markedly improved glycemic control in diet-induced obese mice and leptin receptor deficient *db/db* mice [16]. Thus, Il-22 is a potential novel treatment for metabolic disease.

While a great many preclinical and clinical studies have described changes in circulating gut hormones and bile acids after RYGB [11], so far only 2 studies have addressed changes in circulating Il-22 levels. In the first study by Guida et al., circulating Il-22 levels doubled in 35 pre-diabetic/diabetic patients 6 months after bariatric surgery (18 undergoing RYGB and 17 undergoing sleeve gastrectomy) [17]. Additionally, circulating Il-22 levels were markedly increased in non-obese, RYGB-operated Goto-Kakizaki (GK) rats compared with sham-operated counterparts [17]. More recently, Abdalla et al. showed in a genome-wide association study of 1020 individuals that weight loss following RYGB increases circulating Il-22 levels [18]. Both studies were limited by the fact that weight loss control groups were not incorporated. Further, the potential source of Il-22 was not addressed in these studies, nor was its relationship with improvements in metabolic health after RYGB. To address this gap in knowledge, we re-analyzed portal vein plasma and systemic plasma samples taken from genetically obese and glucose intolerant RYGB-operated Zucker fatty rats compared with sham-operated obese and body weight-matched (BWM) controls [19,20].

## 2 Materials and methods

### 2.1 Experimental animals

Forty-one male Zucker fatty *fa/fa* rats were purchased from Charles River, France, aged 6 weeks. Males were originally selected to avoid the confounds associated with variations in the estrous cycle in females [19]. Rats were individually housed under ambient humidity and a temperature of 22 °C in a 12-h light/dark cycle with free access to tap water and Purina 5008 Lab diet (Purina Mills, USA, 4.15 kcal/g, 16.7% of kcal from fat) unless otherwise stated. Part of the phenotypic data from these animals has previously been reported [19,20]. All experiments were reviewed and approved by the Animal Care Committee of the local government of Unterfranken, Bavaria, Germany (License 55.2–2531.01–72/12).

### 2.2 Experimental groups

Rats were randomly allocated into three groups: the first group received sham surgery (Sham; *n* = 11), the second group received RYGB (*n* = 15), and the third group received sham surgery and were calorically restricted to match the body weight of the RYGB group (BWM; *n* = 15). Surgeries were performed and perioperative care was implemented as previously described [19].

### 2.3 Metabolic phenotyping

Food intake and body weight were measured for 28 days after surgery as previously described [19]. On postoperative day 27, an oral glucose tolerance test (OGTT) was performed at the beginning of the dark cycle. For blood glucose measurements during the OGTT, a small tail vein incision was made in 8-h fasted rats and a drop of blood was directly applied onto a glucometer at baseline and 15, 30, 60 and 120 min after 10 mL/kg body weight ingestion of a 25% glucose solution as previously described [19].

### 2.4 Sample collection

On postoperative day 28, overnight-fasted rats were terminally anaesthetized with isoflurane/oxygen mixture 45 min after a fixed meal of 3 g Purina 5008 diet. Portal vein and inferior vena cava (systemic) blood were collected, and plasma was isolated in tubes containing EDTA as previously described [19]. Whole-wall samples were dissected from a subset of rats (*n* = 5–7 for Sham rats and *n* = 4–5 for RYGB rats – no samples were available for BWM rats due to the retrospective nature of this study) from the duodenum, upper jejunum, lower jejunum, ileum, and colon, washed gently in PBS, snap-frozen in liquid nitrogen and stored at −80 °C.

### 2.5 ELISAs

Due to the retrospective nature of this study, there was limited availability of portal vein (*n* = 11 for Sham rats, *n* = 12 for RYGB rats, and *n* = 15 for BWM rats) and systemic (*n* = 8 for Sham rats, *n* = 7 for RYGB rats, and *n* = 8 for BWM rats) plasma samples. Il-22 was measured in portal vein and systemic plasma samples using a mouse/rat Il-22 Quantikine ELISA kit (R&D systems, #M2200) according to the manufacturer's instructions. The intra-assay CV for this ELISA is 4.4% and the inter-assay CV is 10.5%, with an assay range of 8.2–1000 pg/mL for Il-22. The ELISA for systemic plasma insulin measurements at baseline and 2 h after glucose ingestion during the OGTT was performed as previously described [19].

### 2.6 Real-time qPCR

Total RNA was extracted from gut samples using an Rneasy™ plus micro-kit with on-column Dnase treatment to remove residual genomic DNA (Qiagen), and 1 μg was reverse transcribed using an iScript™ cDNA synthesis kit (Biorad) on a primus 96 thermocycler (Peqlab) set at 55 °C for 40 min. Real-time qPCR was performed with 100 ng cDNA using a MESA GREEN MasterMix Plus for SYBR™ Assay kit (Eurogentec) and 100 pmol forward and reverse primers on a CFX96 touch real-time detection system (Biorad) with an annealing temperature of 60 °C and an elongation temperature of 72 °C (40 cycles). Primer sequences for Il-22 were TTCTCCTCCCAGTTATCAGTTGT for the forward primer and GGTGCGGTTGACGATGTAT for the reverse primer [21]. mRNA expression of Il-22 was determined with the ΔΔ Ct method using *Gapdh* (forward primer: TGGATAGGGTGGCCGAAGTA and reverse primer: AAAGGCGGAGTTACAAGGGG; Eurofins) as the reference gene and expressed relative to the Sham group.

### 2.7 Statistics

Data are presented as mean + S.E.M unless otherwise stated. All statistical tests were performed on GraphPad Prism 8.0 software. Two-tailed, unpaired *t*-test, two-tailed paired *t*-test or one-way ANOVA with Tukey's post-hoc test were applied where indicated. A *P* value of ≤ 0.05 was considered statistically significant.

## 3 Results

**Fig. 1:**
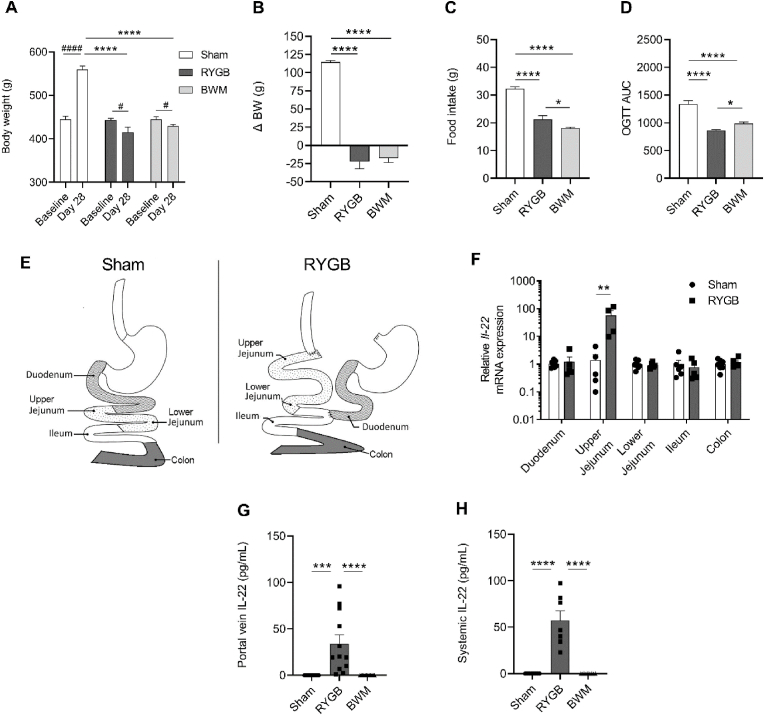
RYGB weight loss-independently induces gut Il-22 release in obese Zucker fatty rats **A.** Body weight in grams (g) at baseline and postoperative day 28, **B.** body weight change in (g) from baseline to postoperative day 28, **C.** food intake (g) at postoperative day 27, and D. area under curve (AUC) of blood glucose during an oral glucose tolerance test (OGTT) at postoperative day 27 in Sham, RYGB and BWM rats (*n* = 10–14). **E.** Schematic diagram showing the gut regions collected for RT-qPCR analysis. **F.** RT-qPCR analysis of *Il-*22 mRNA expression in Sham and RYGB rats (*n* = 4–7). **G.** Portal vein and **H**. systemic plasma levels of Il-22 in Sham, RYGB and BWM rats (*n* = 7–12). Data are presented as mean + SEM in (**A**–**D**) and (**F**–**H**) with individual data points shown in (**F**–**H**). ****P < 0.0001, ***P < 0.001, **P < 0.01 and *P < 0.05 as determined by one-way ANOVA with Sidak post-hoc test in (**A**–**D**) and (**G**–**H**) and with an unpaired, two-tailed *t*-test in **F.**^####^*P* < 0.0001 and ^#^*P* < 0.05 as determined with a paired *t*-test in **A**.

At baseline, rats from each group had similar body weights (Sham: 444.6 ± 7.3g, RYGB: 442.0 ± 5.2g, and BWM: 445.9 ± 5.4g; *P* = 0.91) (Fig. 1A). By study close on postoperative day 28, body weights of RYGB (416.1 ± 11.3g) and BWM (430.2 ± 3.7g) rats were similar (*P* = 0.48) and both significantly less than Sham rats (559.1 ± 8.6g; *P* < 0.0001) (Fig. 1A). Repeated measures *t*-test revealed that weight gain from baseline to postoperative day 28 of Sham rats was significant (*P* < 0.0001), as was the weight loss of RYGB (*P* < 0.05) and BWM (*P* < 0.05) rats during this time-period (Fig. 1A). The absolute change in body weight from baseline to postoperative day 28 for RYGB (−22.64 ± 9.4g) and BWM (−17.5 ± 6.1g) rats was similar (*P* = 0.92) and both were significantly less than that of Sham rats (+114.5 ± 2.5g; *P* < 0.0001) (Fig. 1B). Correspondingly, food intake on postoperative day 27 was lower for RYGB (21.27 ± 1.3g) and BWM (18.1 ± 0.3g) rats compared with Sham rats (32.32 ± 0.7g; *P* < 0.0001) (Fig. 1C). Food intake of BWM rats was also lower than that of RYGB rats (*P* < 0.05) (Fig. 1C), which has been attributed to the higher energy expenditure in the latter [22].

An oral glucose tolerance test was performed on postoperative day 27. Area under the curve (AUC) analysis revealed that RYGB and BWM rats had superior blood glucose clearing capability compared with Sham rats (*P* < 0.0001) (Fig. 1D). Importantly, RYGB rats had superior blood glucose clearing capability compared with BWM rats as well (*P* < 0.05) (Fig. 1D), in line with previous findings from Zucker fatty and Zucker diabetic fatty rats [[8], [9], [10]].

Fig. 1Eprovides a schematic of the gut regions whole-wall samples were collected from for *Il-*22 mRNA expression analysis with real-time quantitative PCR. This revealed that *Il-*22 mRNA expression was approximately 100-fold higher in RYGB rats compared with Sham rats specifically in the upper jejunum (*P* < 0.01) (Fig. 1F).

To determine the impact of RYGB on gut Il-22 release, Il-22 protein was measured by ELISA in portal vein samples. Strikingly, this revealed that Il-22 was detectable in samples from RYGB rats (34.1 ± 9.4 pg/mL), but not from Sham and BWM rats (Fig. 1G). A similar pattern was found in systemic plasma, in which Il-22 was detectable in samples from RYGB rats (46.9 ± 10.5 pg/mL), but not from Sham and BWM rats (Fig. 1H).

**Fig. 2:**
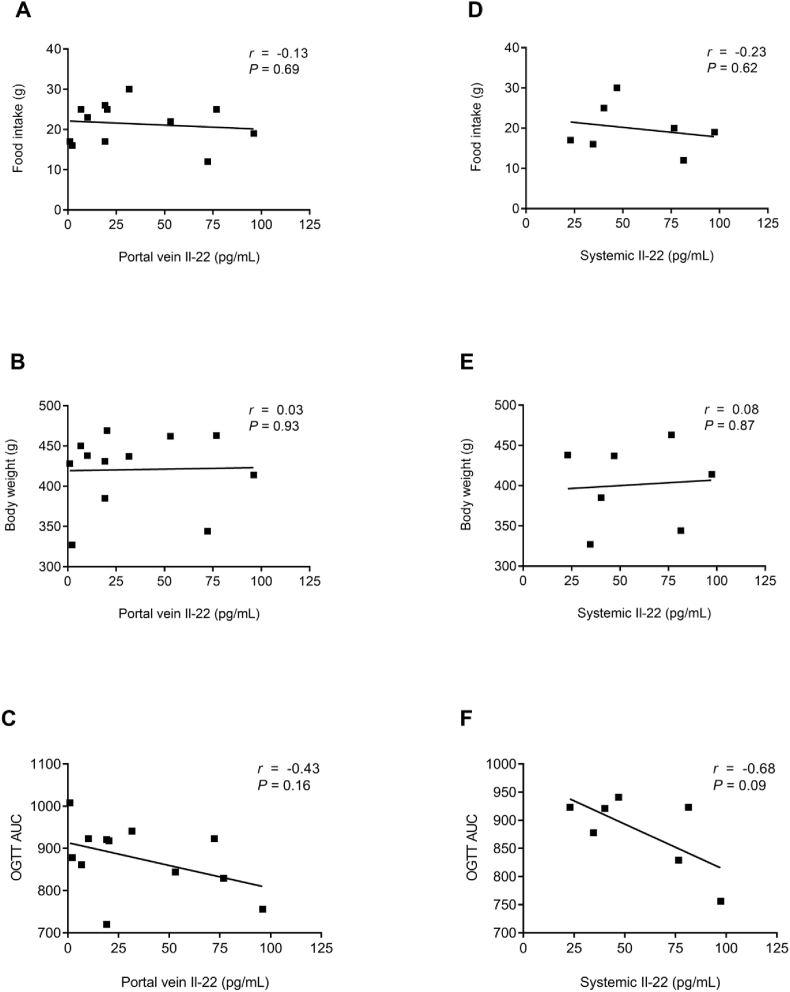
Portal vein and systemic plasma Il-22 levels correlate with improved glycemic control in RYGB rats Pearson correlations between portal vein (A–C) and systemic (D–F) plasma Il-22 levels at postoperative day 28 and food intake at postoperative day 27 (A and D), body weight at postoperative day 28 (B and E) and area under the curve (AUC) of an oral glucose tolerance test (OGTT) at postoperative day 27 (C and F) in RYGB rats (*n* = 12 for portal vein plasma and *n* = 7 for systemic plasma). Solid regression lines indicate least squares fit of data. Statistical significance was determined by two-tailed, unpaired *t*-test.

To determine if the induction of Il-22 release after RYGB is associated with metabolic improvements, correlation analysis was performed. Portal vein plasma Il-22 levels did not show a correlation with food intake (Fig. 2A) or body weight (Fig. 2B) in RYGB rats but did show a trend towards a negative correlation with AUC from the OGTT (*r* = −0.43, *P* = 0.16) (Fig. 2C). Similarly, systemic plasma Il-22 levels did not show a correlation with food intake (Fig. 2D) or body weight (Fig. 2E) in RYGB rats but did show a trend toward a negative correlation with AUC from the OGTT (*r* = −0.68, *P* = 0.09) (Fig. 2F). There was no correlation found between systemic plasma insulin levels at baseline and 2h into the OGTT with portal vein or systemic plasma Il-22 levels (Supplementary Fig. 1).

## 4 Discussion

Identifying the factors that contribute to the weight loss-independent metabolic improvements of RYGB has been challenging [11]. In the present study, we found that mRNA expression of the metabolically beneficial cytokine *Il-22* is markedly increased in the upper jejunum of RYGB rats compared with Sham rats. Strikingly, Il-22 protein was only detectable in portal vein and systemic plasma samples of RYGB rats, and in a manner that correlated with their ability to clear blood glucose in response to an oral glucose challenge. This is consistent with the insulin sensitizing effects of Il-22 in key metabolic tissues like muscle, liver and fat [16]. Further, like RYGB, Il-22 improves glycemic control independently of its effects on food intake [16].

The findings of the present study are in line with those of Guida et al. showing a marked increase in systemic plasma Il-22 levels in non-obese, RYGB-operated GK rats [17]. However, we performed RYGB in obese rats, which is more relevant to the procedure in humans. In addition, we incorporated a body weight-matched control group in our experiments clearly showing that the effects of RYGB on Il-22 release are fully independent of weight loss. One potential mechanism for the increase in gut Il-22 expression and release after RYGB is through the well-established shifts in gut microbiota [23]. Various gut innate lymphoid cells and CD4^+^ T helper cells synthesize Il-22 in response to microbial metabolites such as aryl hydrocarbon receptor (AHR) agonists [24]. One study showed that RYGB increases circulating AHR agonists in humans [25], raising the possibility that the changes in jejunal microbiota postoperatively increases luminal AHR agonist levels to in turn induce gut Il-22 release and improve insulin sensitivity. Indeed, genetically enhancing gut AHR agonist levels in mice improves metabolic fitness through increased gut Il-22 production [26].

Our study has several limitations. Obesity in Zucker fatty rats due to a mutation in the leptin receptor gene does not properly model common obesity in humans. However, leptin does not affect the release of Il-22 from gut immune cells, and the absence of Il-22 in portal vein and systemic plasma samples of Sham rats is most likely explained by their obesity [16]. There was a low number of systemic plasma samples available for Il-22 measurements. Nevertheless, a correlation with glycemic control in RYGB rats could be established. Another limitation of the present study is its observational nature. Experiments on Il-22 deficient mice are needed to prove that Il-22 mediates the weight loss-independent improvements in glycemic control after RYGB. Notably, we previously found that portal vein and systemic plasma levels of the anorexigenic cytokine growth differentiation factor 15 (GDF15) is increased in the same RYGB rats analyzed in the present study, and in a manner that correlates with their body weight and food intake [20]. This raises the possibility that different cytokines may account for distinct metabolic benefits of RYGB. Finally, it would be important to translate the preclinical findings in the present study in future clinical studies. Of particular interest would be to determine if non-responders to RYGB have blunted Il-22 release compared to responders in terms of improvements in glycemic control. If this were to be the case, then Il-22 could potentially serve as an adjunct to RYGB in such patients. Similarly, it would be interesting to determine if Il-22 in combination with dieting has additive or synergistic effects on glycemic control.

## 5 Conclusions

We have presented preclinical evidence showing that RYGB weight-loss independently induces gut Il-22 release in association with improvements in glycemic control, providing further support for the use of this cytokine to treat metabolic disease.

## Funding

The research in this manuscript was funded by the Interdisciplinary Center for Clinical Research of Wuerzburg (IZKF) (grant number Z-3/44 for F.S.) and the 10.13039/501100001659German Research Foundation (DFG) (grant number HA 8213/3–1 for M.K.H., SCHL 1962/7–1 for N.S., and SE 2027/5–1 for F.S.).

## CRediT authorship contribution statement

**Florian Seyfried:** Data curation, Funding acquisition, Investigation, Methodology, Project administration, Resources, Supervision. **Rebecca Springer:** Investigation. **Annett Hoffmann:** Investigation. **Maximilian Gruber:** Investigation. **Christoph Otto:** Methodology, Project administration. **Nicolas Schlegel:** Funding acquisition, Project administration, Resources, Supervision. **Mohammed K. Hankir:** Conceptualization, Data curation, Formal analysis, Funding acquisition, Investigation, Project administration, Resources, Supervision, Validation, Visualization, Writing – original draft, All authors have read and agreed to the publication of this manuscript.

## Declaration of competing interest

The authors declare no conflict of interest.
