# Supplementary material for: Gastric bypass surgery weight loss-independently induces gut Il-22 release in association with improved glycemic control in obese Zucker fatty rats
Source: Metabol Open. 2022 Oct 2;17:100212. doi: 10.1016/j.metop.2022.100212 (PMC10040960; doi:10.1016/j.metop.2022.100212)
Supplement: Multimedia component 1 [file mmc1.docx]

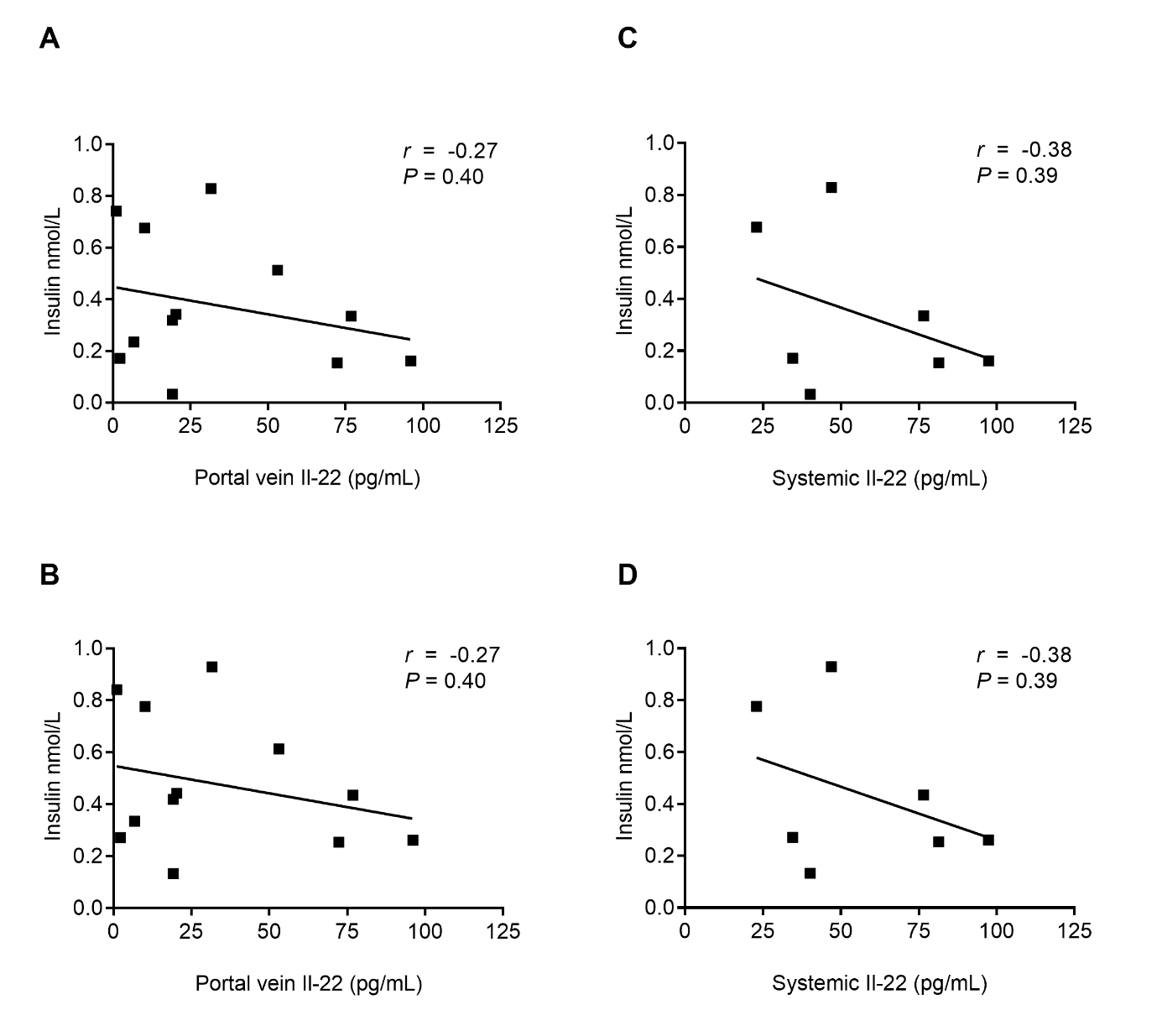


Supplementary Figure 1. Correlations between systemic plasma insulin during the OGTT with portal vein and systemic plasma Il-22 levels in RYGB rats.

Pearson correlations between portal vein (A and B) and systemic (C and D) plasma Il-22 levels at postoperative day 28 with systemic plasma insulin levels during an oral glucose tolerance test (OGTT) at postoperative day 27 at baseline (A and C) and 2 hours after glucose ingestion (B and D) in RYGB rats (*n* = 12 for portal vein plasma and *n* = 7 for systemic plasma). Solid regression lines indicate least square fit of data. Statistical significance was determined by two-tailed, unpaired *t*-test.
